# Hereditary Background in Bone and Soft-Tissue Tumours

**DOI:** 10.1080/135771402753667655

**Published:** 2002-05


### HEREDITARY BACKGROUND IN BONE AND SOFT-TISSUE TUMOURS

### ORAL PRESENTATION

### B22 Osteosarcoma Subtypes in Osteosarcoma Patients with Multiple Primary Malignancies. Can Rare Subtypes be Indicative for Cancer Syndromes Related with Osteosarcoma?

E.I. Hauben[1], J.J. Arends[2], J.P. Vandenbroucke[2], C. van Asperen[2], E. van Marck[3], P.C.W. Hogendoorn[2]
*[1]Stichting PAMM, Eindhoven, The Netherlands, [2]Leiden University Medical Center, Leiden, The Netherlands, [3]Universitair Ziekenhuis, Antwerpen, Belgium*

Although the overall incidence of osteosarcoma is low the occurrence of osteosarcoma in a setting of multiple primary tumours is not infrequent. Association between osteosarcoma and other malignancies is frequently the result of treatment, but can also be the result of genetic predisposition. The aim of our study is to establish the incidence of osteosarcoma associated with other malignancy in the Dutch population and to find out if rare osteosarcoma subtypes occur in higher frequency in patients with secondary malignancies and who are suspected to have a genetic predisposition to malignancy.
*Methods:* Of all patients with the diagnosis of osteosarcoma registered between 1975 and May 2000 in the Dutch National Pathology Register, all patients were selected who had an other malignancy before or after their diagnosis of osteosarcoma. In a second step only those patients were retained for further study that had a history of retinoblastoma or a history of malignancy before the age of 45. Of these patients the histological material was reviewed and osteosarcomas were subtyped as common, chondroblastic, fibroblastic, anaplastic, telangiectatic, osteoclast rich or small cell. As a control 570 patients entered in the database of the European Osteosarcoma Intergroup were used.
*Results:* In total 1145 patients had a diagnosis of osteosarcoma, 65 had a history of multiple primary tumours. 7 of these patients were rejected for further analysis, 2 were retinoblastoma patients and 22 had their malignancies before the age of 45. 47% of these patients had an osteosarcoma subtype other than common as opposed to 29% in the control group. If only those patients were selected in which there was no relation with treatment and second malignancy, 90% had subtypes other than common.
*Conclusion:* a non-common subtype of osteosarcoma may be predictive for a patient to be a member of a cancer syndrome family.

### B23 Gene and Protein Expression Profiling in Fibroblasts Carrying a Li-Fraumeni p53 Germline Mutation that Induces Chemoresistence to Doxorubicin

L. Sangiorgi[1], M.A. Cerone[2], S. Soddu[2], G.A. Gobbi[1], E. Lucarelli[1], F. Scrimieri[1], V. Maini[1], A. Brach del Prever[3], P. Picci[1], L.J. Helman[4]
*[1]Istituti Ortopedici Rizzoli, BOLOGNA, Italy, [2]Regina Elena Cancer Institute, Rome, Italy, [3]University of Turin, Turin, Italy, [4]NCI, NIH, Bethesda, MD, USA*

*Objective of the study:* The study of gene expression profiling (with cDNA microarrays) and protein expression (by co-immuno precipitation) help in the identification of candidate genes and proteins involved in oncogenic pathways in the search of novel therapeutic targets.
*Methods:* In a phenotypic Li-Fraumeni family, a germline p53 mutations at codon 220 (amino acid change from tyrosine to serine) was identified. We generated by site directed mutagenesis a plasmid encoding the p53SER220 mutation (pLp53-S220) and transfected fibroblasts from p53 -/- mice (F10) with this vector. Drug sensitivity (assessed by IC50 value) of the fibroblasts carrying the mutation was evaluated for doxorubicin, cisplatin and 5-florouracil. Fibroblasts carrying the p53SER220 mutation showed a selective resistance for doxorubicin. We treated with doxorubicin the p53SER22, p53 wild type and p53 -/- fibroblasts and we evaluated the gene expression profiles of these cells at different time points. Moreover, we performed a co-immuno precipitation with a p53 antibody in cellular lysate of p53 wild type and p53SER220 fibroblasts also treated with doxorubicin to evaluate the protein expression profiles at different time points.
*Results:* Distinct patterns of gene expression for the three different fibroblasts were identified. Additionally, lists of doxo-dependent and doxo-independent activated genes were determined. Analysis of these gene lists pinpoint a number of signal transduction and cell cycle genes differentially expressed. Co-immuno precipitation experiments resulted in the presence of additional bands 24 h after doxorubicin treatment when the p53SER220 fibroblasts were compared with the wild-type cells and these bands could identify proteins involved in the chemoresistance.
*Conclusion:* Several genes and protein are expressed according to the different p53 status after doxorubicin treatment. These studies may evolve in development of novel therapeutic strategies.

### B24 Somatic and Germinal Alteration of the TP53 Gene in Pediatric Osteosarcoma

A. Pati o, E. 1 Sotillo, M. Zalacain, L. Gárate, M. San-Julian, E. Noain, L. Sierrasesumaga
*University Clinic of Navarra, Pamplona, Spain*

Paediatric osteosarcoma (OS) is a puzzle of genetic alterations in genes involved in the arrest of the cell cycle in response to DNA damage. Alteration of the TP53 gene has been detected in sporadic and in hereditary OS in the context of the Li-Fraumeni Syndrome.
*Objective:* To analyse the alteration of the TP53 gene in Spanish paediatric OS both at the germinal and somatic levels.
*Methods:* Blood samples were obtained from 76 OS (42 males/34 females, median age at diagnosis 13.13±3.16 years), 136 healthy volunteers and paired tissues were available from 41 of the patients. Loss of heterozygosity was tested by the analysis of P53IVS1, and mutation by PCR-DGGE and sequencing.
*Results:* TP53 alteration was detected in 42.9% of the tissues and in 5.3% of blood samples (Table I). Mutations were nucleotide changes except two cases with frameshift mutations. Table I. Features of the OS patients with TP53 germline mutations. TP53 mutation Necrosis/subtype Survival/*EFS Other findings
#1Codon 250 (CCC-TTC) 20%/Chondroblastic 54/0 Died of secondary ALL
#2Codon 223 (G deletion) 80%/Osteoblastic145/145 Long term remission
#3Codon 196 (CGA-TGA) 90%/Chondroblastic 84/0 Died of disease. Mother and grandfather died of leukaemia and colon respectively
#4Codon 132 (AAG-AAC) 40%/Chondroblastic11/-Still in treatment
*EFS: Event-free survival in months.
The median age at diagnosis of patients with mutated TP53 (15.8 years) was statistically higher (p=0.038) than that of patients with normal TP53 (12.8 years).
*Conclusion:* Alterations of TP53 are present in about half of OS tumour samples, confirming the outstanding role of this gene in tumourigenesis. Four of our patients were germline carriers of TP53 alterations, while none of the healthy controls had TP53 mutation. The difference in the age at diagnosis depending on

TP53 mutation status has also been detected by other authors, reflecting the existence of different subsets of OS patients that should be further investigated.

**B25 Chromosome 9 Alterations and p16 Expression in Central Chondrosarcomas**

H.M. van Beerendonk[1], L.B. Rozeman[1], A-M. Cleton-Jansen[1], R. Sciot[2], A.H.M. Taminiau[3], J.V.M.G. Bovée[1], P.C.W. Hogendoorn[1]
*[1]Dept. of Pathology, Leiden University Medical Center, Leiden, The Netherlands, [2]Dept. of Pathology, Leuven University, Leuven, Belgium, [3]Dept. of Orthopaedic Surgery, Leiden University Medical Center, Leiden, The Netherlands*

*Introduction:* Chondrosarcomas are characterised by neoplastic growth of cartilage forming tumour cells. The majority (75%) arises centrally, in the medullar cavity, while a minority develops peripherally secondary to an osteochondroma. We previously investigated DNA-ploidy and loss of heterozygosity (LOH) at loci harbouring the EXT-genes (implicated in hereditary multiple exostoses), the EXT-like genes, and at 9p21, 13q14, 17p13 and chromosome 10 in 12 central chondrosarcomas. Only 3 cases exhibited LOH, with 9p21 involved in all three. At 9p21 the p16 tumour suppressor gene is located. Previous studies showed in seven central chondrosarcomas an abnormal karyotype, 5 of which involved chromosome 9. Questions are the cell cycle inhibitors CDKN2A/p16 or CDKN2B/p19, located at 9p21, involved in chondrosarcoma tumourigenesis?
*Methods:* p16 immunohistochemistry was performed on formalin-fixed paraffin-embedded tissue of 60 cases of central chondrosarcoma to estimate the effect of 9p21 alterations on p16 protein expression. Loss of heterozygosity (LOH) analysis was performed in the region of p16 on 52 central chondrosarcomas. SSCP analysis was done on 48 cases for p16 and p19. Methylation of the p16 gene promoter was studied by methylation specific PCR (MSP).
*Results:* The involvement of genes located at chromosome 9, especially the 9p12–22 region, is suggested both by LOH, p16 immunohistochemistry and cytogenetic studies. Loss of expression of the p16 protein was found in 19.7% of the cases. LOH was found on 9p in 47% of the informative cases. SSCP analysis did not reveal mutations in the p16 or p19 genes in any of the tumours. MSP analysis showed that in some tumours methylation of the p16 promoter occurs when p16 protein expression is absent or decreased.
*Conclusion:* our results show that inactivation of p16 protein expression occurs in central chondrosarcoma and may be caused by LOH and promoter methylation.

**B26 Identification and Clinical Validation of Candidate Target Genes from the Amplified Region on Chromosome Arm 17Q in Malignant Peripheral Nerve Sheath Tumours**

S. Smeland[1], R.I. Skotheim[1], O. Monni[2], J.M. Nesland[1], B. Bjerkhagen[1], A.E. Stenwig[1], F. Mertens[3], O-P. Kallioniemi[4], A. Kallioniemi[4], R.A. Lothe[1]
*[1]The Norwegian Radium Hospital, OSLO, Norway, [2]Biomedicum Helsinki, Helsinki, Finland, [3]University Hospital, Lund, Sweden, [4]NHGRI, National Institutes of Health, Bethesda, USA*

Patients with neurofibromatosis type I are predisposed to acquire malignant tumours, including malignant peripheral nerve sheath tumours (MPNSTs). By genome wide DNA copy number analyses by comparative genomic hybridisation (CGH) we discovered that most MPNSTs have extra copies of the distal part of the long arm on chromosome 17 (Lothe et al., Cancer Res. 56(20):4778–81 1996). Others have later confirmed this finding. In order to identify the overexpressed target gene(s) responsible for the selective advantage of this genomic amplification in tumours, we submitted a series of 14 MPNSTs to a detailed gene expression profiling focused on chromosome 17. The custom made chromosome 17-specific cDNA microarrays contained 636 transcribed sequences, including all known genes as well as hundreds of expressed sequence tags from chromosome arm 17q. This led us to the identification of several differentially- and overexpressed genes, some of which we consider as candidates for driving the genomic amplification. The five mostly overexpressed genes/transcripts on chromosome arm 17q were AP2B1, GCN5L2, TIMP2, TOP2A, and an expressed sequence tag (Image clone ID 1097923). Candidate genes are currently under clinical validation in a cohort of 41 patients admitted to the Norwegian Radium Hospital the past 20 years. Protein expressions are evaluated by immunohistochemistry on a tissue microarray made from qualified tumour areas from the archival blocks.

**B27 Association Between the Occurrence of Breast Cancer and Cartilaginous Tumours: Phenotypic Characterization of a Hitherto Unrecognized Potential Hereditary Trait**

A.M.Cleton-Jansen, M. Timmerman, I. Briaire-de Bruijn, M. van de Vijver, C. van Asperen, L.C.J.M. van den Broek, H.M. Kroon, P.C.W. Hogendoom
*Leiden University Medical Center, Leiden, The Netherlands*

*Introduction:* Recently we documented a strong association between the occurrence of cartilaginous tumours (enchondroma, chondrosarcoma) and breast cancer in the same patient, using a nation-wide case-control study. This study revealed an odds ratio of 7.62 for a potential association of breast and cartilaginous tumours, pointing statistically strongly towards a genetic trait. This is furthermore corroborated by the age of onset in patients with breast cancer as the first tumour, which is about 10 years earlier than breast cancer in the general population.
*Objective:* We have investigated clinical and phenotypic properties of the breast- and the cartilaginous tumours in patients with both tumour types to identify a possible characteristic spectrum for this hereditary trait.
*Methods:* Using the national pathology database the tissue blocks of all patients reported to fulfil the associated tumours mentioned were retrieved. Reported diagnoses were reviewed; breast cancer specimens were classified and histologically. In addition the cartilaginous tumours were analysed with emphasis on the central versus peripheral localisation in the skeleton as previous studies proved a different molecular mechanism to be operative in the different subtypes. Expression of p53, Bcl2, Her2-neu, p16, p21 oestrogen and progesterone receptor and E-cadherin was determined by immunohistochemistry.
*Results and Conclusion:* No case of chondrosarcoma nor enchondroma was reported in the Dutch BRCA1 and BRCA2 database, pointing to a trait which is different from the aforementioned breast cancer syndromes. Remarkably all cartilaginous tumours are of one common histological subtype being centrally localised whereas no peripheral cartilaginous tumour was registered. Furthermore the cartilaginous tumours showed a significantly higher p16 and p21 staining. The breast tumours were histologically heterogeneous with varying differentiation grade. Characteristics of the breast cancers: 10 years earlier age of onset, higher mitotic count, less lymph infiltrate, high ER staining and high p53 staining.

### POSTER PRESENTATION

### B28 Aneurysmal Bone Cyst: A Heriditary Disease?

A.L. Leithner[1], F. Machecek Jr.[2], O.A. Haas[3], S. Lang[4], P. Ritschl[2], R. Kotz[4], R. Windhager[1]
*[1]Karl Franzens University, Graz, Austria, [2]Orthopedic Hospital Gersthof, Vienna, Austria, [3]St. Anna Children's Hospital, Vienna, Austria, [4]Vienna University Hospital, Vienna, Austria*

*Object of study:* Recent genetic and immunohistochemical studies propose that primary aneurysmal bone cyst is a tumour and not a reactive tumour-simulating lesion. Furthermore, chromosomal analysis and five reported familial cases of this osteolytic bone lesion propose a hereditary factor in a presumably multifactorial pathogenesis.
*Methods:* The authors contacted 133 patients with this disease. 66 females and 66 males (median age 14 years; range 2–66) were asked if they knew of the occurrence of bone lesions at other family members. If the answer was positive, histology, radiology, and the patient's history were reviewed.
*Results:* 103 patients (77%) denied, 25 patients (19%) were lost to follow up, and 5 patients (4%) gave evidence for other bone lesions in the family – two patients were father and son (this case has been reported in 1998), another patient knew of a giant cell tumour G1 of an uncle's distal tibia, the father of a further patient had an asymptomatic small cystic defect in the right proximal femur, and another patient knew that forty years ago her father underwent surgery for a cystic lesion of the palate.
*Conclusion:* These data indicate that a predisposing genetic defect could be part of a multifactorial pathogenesis of aneurysmal bone cyst. Furthermore, the coincidence of an aneurysmal bone cyst and a giant cell tumour in one family rises questions for a stronger context between those two, sometimes strikingly similar lesion.

### B29 Expression Profiling of Osteosarcoma Biopsies and Metastases with cDNA Microarrays

L. Sangiorgi[1], G.A. Gobbi[1], E. Lucarelli[1], F. Scrimieri[1], V. Maini[1], P. Picci[1], L.J. Helman[2]
*[1]Istituti Ortopedici Rizzoli, Bologna, Italy, [2]NCI, NIH, Bethesda, MD, USA*

*Objective of the study:* Expression profiling with cDNA microarrays offers the possibility to determine global patterns of gene expression tumour specimens which can be used for diagnostic classification, the identification of subsets as well as the elucidation of oncogenic pathways and novel therapeutic targets.
*Methods:* We have explored the potential of applying this technology to osteogenic sarcoma. 14 primary osteosarcomas and 8 osteosarcoma pulmonary metastases were analysed by hybridisation to cDNA microarrays. For comparison, five Ewing's sarcoma specimens, and normal lung tissue were also analysed.
*Results:* We determined that high quality results could be obtained from osteosarcoma specimens despite the presence of extracellular matrix material in these specimens. A distinct pattern of gene expression for osteosarcoma was readily identified. This osteosarcoma specific gene expression signature was detectable in pulmonary metastases. Additionally, lists of genes, which discriminate osteosarcoma and Ewing's sarcoma, were developed. Inspection of these gene lists identifies a number of signal transduction and cell cycle genes utilised differentially by these two cancers.
*Conclusion:* Several genes expressed by osteosarcoma have potential for development as targets for immunohistochemical detection for diagnostic purposes.

### B30 High Quality RNA Isolation from Tumours with Low Cellularity and High Extracellular Matrix

J.J. Baelde[1], A-M. Cleton-Jansen[1], H.M. van Beerendonk[1], M. Namba[2], J.V.M.G. Bovée[1], P.C.W. Hogendoorn[1]
*[1]Dept Pathology, Leiden University Medical Center, Leiden, The Netherlands, [2]Dept. of Cell Biology, Institute of Molecular and Cellular Biology, Okayama University Medical School, Shikata, Japan.*

*Introduction:* High quality RNA isolation from cartilaginous tissue is considered difficult due to relative low cellularity and the abundance of extracellular matrix rich in glycosaminoglycans and collagen. Given the growing interest and technical possibilities to study RNA expression at a high throughput level research on tissue bearing these characteristics is therefore hampered.
*Methods:* We present a robust protocol combining two RNA isolation procedures based on a combination of trizol and RNA specific columns that has been developed to obtain high molecular weight RNA from fresh frozen and stored tissue of normal cartilage and cartilaginous tumours. Using this method RNA was isolated from normal cartilage, peripheral and central chondrosarcoma.
*Results:* RNA per mg tissue. RNA isolated with this method was stable and of high molecular weight. RNA samples from normal cartilage and from two chondrosarcomas isolated using this method were applied successfully in cDNA microarray experiments; The yields ranged from 0.1–0.5 The number of genes that give interpretable results was in the range of what is expected when compared with microarray results obtained on chondrosarcoma cell line RNA. Signal-to-noise ratios were good and differential expression between tumour and normal cartilage was detectable for a large number of genes.
*Conclusion:* With this newly developed isolation method high quality RNA can be obtained from low cellular tissue with high extracellular matrix component. These procedures can be applied to other tumour material as well.

### B31 Role of the MET/HGF Receptor in Proliferation and Invasive Behaviour of Osteosarcoma

R Ferracini[1], N Coltella[1], MF Di Renzo[1], C Manara[2], L Trusolino[1], K Scotlandi[2]
*[1]IRCC, Candiolo (Torino), Italy, [2]Istituti Ortopedici Rizzoli, Bologna, Italy*

The hepatocyte growth factor (HGF/SF) receptor Met regulates mitogenesis, motility and morphogenesis in a cell type-dependent fashion. The signal transduction pathway activated by Met after binding of HGF and dimerization of the receptor involves transphosphorylation of the intracellular domain of Met and leads to the activation of multiple pathways accounting for the complex invasive growth response.
*Methods:* We analysed the effect of the aberrantly expressed Met receptor gene in osteosarcoma cell lines characterising the downstream-activated pathways and phenotypic changes induced by Met activation. Five cell lines derived from human osteosarcomas were tested: U-2OS, MG-63, Saos-2, IOR/OS9 and IOR/OS10. We evaluated the levels of MAPK and PKB/Akt activation following stimulation with HGF by Western blot analysis. For cell growth assay, cells were starved in medium devoid of serum and growth factors for 48 h, detached and plated in 24-well plates in a serum-free medium supplemented with different concentration of HGF for 48 hours. Cell number was estimated after staining with Crystal Violet by a colorimetric assay. A motility assay was performed evaluating the migration of the cells through a polycarbonate filter (Transwell).

*Results:* All the cell lines tested expressed high levels of the MET proto-oncogene and two of them (MG-63 and IOR/OS10) co-expressed HGF. Four cell lines (U-2OS, Saos-2, IOR/OS9 and IOR/OS10) showed the activation of the MAPK cascade suggesting a common proliferative role of the HGF. Two lines (U-2OS, Saos-2) showed activation of PKB/Akt, which is known to be involved in migration mediated by HGF receptor. These data are confirmed by proliferation and motility assay in vitro.
*Conclusion:* These data suggest that HGF activates both the mitogen and motogen machinery in osteosarcoma cells, promoting the malignant behaviour of these cells at different steps. Inhibition of the paracrine/autocrine HGF/Met circuit may represent a promising target for innovative therapies in osteosarcoma.

### B32 Telomerase Activity and Telomere Length in Pediatric Bone Tumours

E. Sotillo, M. San-Julian, E. Noain, L. Sierrasesúmaga, A. Pati o
*University Clinic of Navarra, Pamplona, Spain*

Telomeres are structures at the end of eukaryotic chromosomes that are shortened with each cellular division. Telomerase is the ribonucleoprotein that adds telomeric DNA compensating for telomere loss, and is expressed in about 85–90% of all tumour types. Bone tumours account for 7% of all childhood malignancies in Spain: 4% are osteosarcomas (OS) and 3% Ewing's sarcomas (ES).
*Objective:* To analyse the presence of telomerase activity (TA) and the TRF length (Telomeric Restriction Fragments) in primary and metastatic tissues from Spanish paediatric OS and ES patients.
*Methods:* TA was determined by TRAP (Telomeric Repeat Amplification Protocol) analysis in tumour tissue samples obtained from OS (9 primary tumours, 8 metastases) and ES patients (8 primary tumours, 5 metastases). The TRF length was measured by Southern Blot in the same tissue samples.
*Results:* Telomerase Activity (TA) was detected in 84.5% (11/13) of the metastatic tissues and in 11.7% (2/17) of the primary tumours ($p<0.001$) (Table I).
Table I. Telomerase activity in bone tumour tissues.
Sample typeNumber analysed/Number with TA +
OS Primary tumour 9/1
Metastases 8/6
ES Primary tumour 8/1
Metastases 5/5
Bone Tumours Primary tumour 2/17
Metastases 11/13
The mean TRF length of samples that lacked telomerase activity (11.6 Kb) was statistically longer ($p=0.041$) than that of samples with detectable telomerase activity (8.7 Kb).
*Conclusion:* TA is not responsible for the telomeric maintenance in the initial steps of the carcinogenesis of paediatric bone tumours, but is essential for achieving the full malignant phenotype and metastatic capacity. Primary bone tumours must use any Alternative Lengthening Telomere mechanisms, which are related to longer TRFs. A possible hypothesis is that a scarce numbers of tumoural cells that posses telomerase activity are those that generate the metastatic nodules.

### B33 Protease Inhibitors as Prognostic Factors in High Risk Soft Tissue Sarcomas (STS)

M.S. Beans, G. Magagnoli, F. Ponticelli, L. Pazzaglia, G. Gamberi, P. Ragazzini, P. Picci
*Istituti Ortopedici Rizzoli, Bologna, Italy*

Matrix metalloproteinases (MMPs) degrade components of the extracellular matrix and are implicated in tissue remodelling and tumour infiltration. Tissue inhibitors of metalloproteinases (TIMPs) inhibit enzymes of the MMP family and preserve stromal integrity, thus inhibiting tumour migration. An unbalance in this proteolytic activity may contribute to tumour invasiveness and metastasis. The present study evaluated MMP2 and -9 tissue enzymatic activity as well as the expression and serum level of their inhibitors TIMP1 and -2, in selected patients with high risk STS, to evaluate the alterations of proteolytic cascade in malignancy progression.
*Methods:* The level and distribution of MMP2, MMP9, TIMP1 and TIMP2 expression were evaluated in 69 biopsies of primary STS including: 15 high grade liposarcomas, 19 synovial sarcomas, 7 high grade MPNST and 28 high grade MFH. The study was carried out by immunohistochemistry and zymography. To test plasma TIMP1 and -2 concentration, blood samples were collected from 53 patients before treatment and measured with enzyme-linked immunoabsorbent assay (ELISA). Histological types included 20 liposarcomas, 13 synovial sarcomas and 20 MFH. 58 healthy subjects were included as control group.
*Results:* By zymography analysis, 52% and 49% of the 69 tumours had significant enzymatic activity of MMP2 and MMP9 respectively. TIMP2 expression was inversely correlated to poor prognosis in terms of disease free survival and overall survival ($p<0{,}003$, $p<0{,}006$ respectively). In the metastatic group, 78% patients lacked TIMP2 expression versus 43% in the disease free patients. Plasma concentration of TIMP1 and -2 in 53 STS patients revealed significantly lower levels compared to controls.
*Conclusion:* The Results of this study showed that TIMP1 and -2 are involved in the progression of high grade STS.

### B34 Primary Malignant Tumours of Bone Following Previous Malignancy

J.T. Patton, S. Sommerville, R. Grimer
*Royal Orthopaedic Hospital, Birmingham, United Kingdom*

The purpose of this study is to emphasise the necessity for caution in assuming the diagnosis of a metastasis when a solitary bone lesion is identified following a prior malignancy. Bone lesions occurring in patients who have previously had a malignancy are generally assumed to be a metastasis from that malignancy. We reviewed 60 patients with a previous history of malignancy, who presented with a bone lesion that was subsequently found to be a different primary sarcoma of bone. These second malignancies occurred in three distinct groups of patients.

1. Patients with original tumours well known to be associated with second malignancies (5%)
2. In patients whose second malignancies were likely to be due to the previous treatment of their primary malignancy (40%)
3. In patients in whom there was no clearly defined association between malignancies (55%)

Inappropriate biopsy and treatment of primary bone sarcomas compromises limb salvage surgery and can affect patient mortality. We would advise referral of any aggressive solitary bone lesion to a regional bone tumour service for further assessment and biopsy rather than to assume the lesion is a metastasis.

### B35 Protease Inhibitors as Prognostic Factors in High Risk Soft Tissue Sarcomas (STS)

A. Balladelli, M.S. Benassi, G. Magagnoli, F. Ponticelli, L. Pazzaglia, G. Gamberi, P. Ragazzini, P. Picci
*Istituti Ortopedici Rizzoli, Bologna, Italy*

Matrix metalloproteinases (MMPs) degrade components of the extracellular matrix and are implicated in tissue remodelling and tumour infiltration. Tissue inhibitors of metalloproteinases (TIMPs) inhibit enzymes of the MMP family and preserve stromal integrity, thus inhibiting tumour migration. An unbalance in this proteolytic activity may contribute to tumour invasiveness and metastasis. The present study evaluated MMP2 and -9 tissue enzymatic activity as well as the expression and serum level of their inhibitors TIMP1 and -2, in selected patients with high risk STS, to evaluate the alterations of proteolytic cascade in malignancy progression. The level and distribution of MMP2, MMP9, TIMP1 and TIMP2 expression were evaluated in 69 biopsies of primary STS including: 15 high grade liposarcomas, 19 synovial sarcomas, 7 high grade MPNST and 28 high grade MFH. The study was carried out by immunohistochemistry and zymography. To test plasma TIMP1 and -2 concentration, blood samples were collected from 53 patients before treatment and measured with enzyme-linked immunoabsorbent assay (ELISA). Histological types included 20 liposarcomas, 13 synovial sarcomas and 20 MFH. 58 healthy subjects were included as control group. By zymography analysis, 52% and 49% of the 69 tumours had significant enzymatic activity of MMP2 and MMP9 respectively. TIMP2 expression was inversely correlated to poor prognosis in terms of disease free survival and overall survival ($p<0{,}003$, $p<0{,}006$ respectively). In the metastatic group, 78% patients lacked TIMP2 expression versus 43% in the disease free patients. Plasma concentration of TIMP1 and -2 in 53 STS patients revealed significantly lower levels compared to controls.
*Conclusion:* The Results of this study showed that TIMP1 and -2 are involved in the progression of high grade STS.

**B36 Using Spectral Karyotyping and Array-Based CGH to Detect Specific Genomic Amplification in Rhabdomyosarcoma**

Y. Kollender[1], M. Goldstein[2], A. Bar-Shira[2], H. Rennert[2], R. Shomrat[2], J. Issakov[3], A. Orr-Urtreger[2], I. Meller[1]
*[1]Nat. Unit of Orthopedic Oncology, [2]The Genetics Institute and [3]Pathology Institute, Tel Aviv Sourasky Medical Center, Tel Aviv, Israel*

Rhabdomyosarcoma (RMS) is histologically divided into two main subtypes: embryonal (ERMS) and alveolar (ARMS), each associated with different genetic changes. ARMS is characterized by the fusion of FKHR at 13q14 with either PAX3 at 2q35, or, less commonly, with PAX7 at 1p36, while no consistent chromosomal abnormalities are observed in ERMS. To identify novel genetic changes associated with RMS, cytogenetic and array-based comparative genomic hybridisation (array-based CGH) analyses were employed on eight pathologically-diagnosed RMS tumours. Of the 3 ERMS specimens examined, 2 demonstrated a normal karyotype, while the third sample displayed changes in several chromosome including 2, 7 and 8. In ARMS, structural rearrangements were detected in 4 of the 5 tumours tested. Involvement of 13q14 region was detected in 3 cases, but only one case showed a characteristic t(2;13), in addition to other aberrations. The other 2 cases displayed ins(13;13) (q14;q1?) in a complex karyotype, and a t(13;20) (q14;q13.3), in addition to a large number of double minutes (dmins), respectively. Spectral karyotyping analysis identified the dmins origin as chromosome 13, while the dmins detected in the last tumour as a sole cytogenetic aberration, were classified as derived from chromosome 1. Array-based CGH analysis of 57 genes commonly amplified in cancer, detected a single co-amplification of GLI (2.5-fold) and SAS/CDK4 (3.2-fold) in the ARMS specimen harbouring the t(13;20) and dmins(13).
*Conclusion:* This data demonstrates that using a combination of advanced molecular and cytogenetic techniques, namely SKY and array-based CGH, allowed us to better characterize some of the genetic changes which may contribute to RMS tumourigenesis, in particular, the dmins and gene amplification events.

**B37 Molecular Karyotyping Approach for the Characterisation of Soft Tissue Tumours.**

K. Szuhai, M. Yszenga, J. Knijnenburg, H.J. Tanke, P.C.W. Hogendoorn, C. Rosenberg
*Leiden University Medical Center, Leiden, The Netherlands*

Soft tissue tumours constitute a heterogeneous and complex group of mesenchymal lesions. Chromosomal analysis and molecular cytogenetic approaches had a great impact on the classification and diagnosis of these tumours. However, because of the lack of molecular markers, it is still difficult to discern in a number of tumour types benign lesions from malignant ones based purely on histology. We set up a complex molecular approach to analyse new cases by using a multicolour FISH (COBRA FISH) approach and comparative genomic hybridisation (CGH) for genome-wide screening and determination of alteration of the tumour genome. Multifluorescent in situ hybridisation using whole chromosome painting probe sets may help to pinpoint recurrent aberration, which would improve the (sub)classification of different entities. Complex chromosomal rearrangements are further characterised by hybridisation-based staining of pan-centomeric/telomeric and subtelomeric sequences. Candidate regions are analysed by high-resolution BAC/PAC hybridisations on small or large-scale arrays to determine new genes involved in the disease. The detected rearrangements are confirmed on archived materials by using FISH or PCR based approaches. So far we have analysed more than 40 new cases of various tumour types and the approach is illustrated with representative cases.

**B38 Growth Hormone Gene Expression in Canine Osteosarcoma**

J. Kirpensteijn, E. van Garderen, E.P.M. Timmermans-Sprang, G.R. Rutteman, J.A. Mol
*Utrecht University, Utrecht, The Netherlands*

*Introduction:* Growth hormone (GH) and Insulin-like growth factor-I (IGF-I) play an important role in bone and osteosarcoma (OS) formation. Local GH gene expression was present in 25% of canine OS (1). The purpose of this study was to evaluate if other important growth promoting factors are present in canine OS and to evaluate if local GH gene expression will affect disease progression in canine OS.
*Methods:* We screened 60 bone tumours for local gene expression of GH, GH receptor (GHR), IGF-I, and insulin-like growth factor binding protein-5 (IGFBP-5), and venous plasma GH level. Multivariate Cox regression analysis was performed to evaluate if the local expression of these factors may affect the clinical outcome of dogs with OS.
*Results:* Local expression of GH, GHR, IGF-I and IGFBP-5 genes was demonstrated in canine OS. There was a significant difference in expression of the GH gene, but not of the GHR, IGF-I and IGFBP-5 gene between high and low-grade tumours. No significant correlation was found between GH expression and expression of any of the other genes or with the central venous plasma GH level. Local expression of the GH gene was associated with a higher hazard ratio for a decreased disease free interval and survival time in dogs with OS.
*Conclusion:* This research substantiates earlier results that canine OS express the GH gene on a local level. The local production of GH most likely plays a role in the growth of neoplastic bone lesions through factors that are common in normal bone formation in the dog, and has a negative effect on survival data.

**B39 Telomere Length is Related to Survival in Pediatric Patients with Bone Tumours Undergoing Chemotherapy Treatments**

E. Sotillo, M. San-Julian, E. Noain, L. Sierrasesúmaga, A. Pati o
*University Clinic of Navarra, Pamplona, Spain*

Telomeres are structures at the end of eukaryotic chromosomes that are shortened with each cellular division, being one of the limiting factors for cellular proliferation. Most of the chemotherapy treatments are designed to act against immortal cells, which have shorter telomeres due to their higher proliferative rates.
*Objective:* To compare the TRF (Telomeric Restriction Fragments) length of blood samples from paediatric osteosarcoma (OS) and Ewing's sarcoma (ES) patients before and after the chemotherapy treatments.
*Methods:* Blood samples were obtained from 15 OS and 18 SE patients at the beginning as well as after the chemotherapy cycles. The TRF length was measured by Southern Blot.
*Results:* TRF length at the beginning of treatment was statistically shorter in patients with survival periods >2 years, compared to those patients who died within 2 years after diagnosis (p=0.032). This difference was conserved when the survival period considered was increased to 5 years (p=0.005). The TRF length of samples at the beginning and at the end of treatment was statistically different in patients with more than either 5 or 2 years of survival, opposite to those patients who died within 2 years after completion of chemotherapy, whose telomeres had the same length at the beginning and after treatment (Table I).
Table I: Difference between the TRF length at the beginning and after treatment in relationship to survival.
Survival Number of samples, Difference in TRF length
< 2 years 90.9 Kbp=0.041
2–5 years 130.5 Kbp=0.046
> 5 years 110.1 Kbp=0.563
*Conclusion:* Bone tumour patients that have shorter TRFs lengths at the beginning of chemotherapy have longer survival rates than those patients with longer TRFs. The difference between the TRF length at the beginning and at the end of treatment seems to be related to survival in bone cancer paediatric patients undergoing chemotherapy.

**B40 Multiple Primary Malignancies in Patients with Soft Tissue Sarcomas**

O. Merimsky, J. Issakov, Y. Kollender, I. Schwartz, J. Bickels, G. Flusser, M. Inbar, I. Meller
*Tel Aviv Sourasky Medical Center, Tel Aviv, Israel*

Modern cancer treatment has substantially increased the survival of patients with various malignancies. One of the late sequelae of a successful treatment is the development of a second malignant tumour. However, in many cases of second primary cancers, exposure to chemotherapy or radiation therapy is not evident, and it should be postulated that the putative mechanism for the development of the second-tumour is different. Retrospective search of data files of 610 patients with soft-tissue or bone-sarcomas were treated by our team of Musculoskeletal-Oncology from January 1995 through December 1999 was performed. Out of 375 patients with STS, 28 (7.5%) developed other malignant neoplasms either before or after its diagnosis. STS as the first tumour occurred in 14 patients. The second-tumour-types included mainly STS and renal-cell-carcinoma. The interval of time between the diagnosis of the STS and the second malignancy was 0 to 21 years. Three patients developed a third primary tumour within 0–3 years after the diagnosis of the second tumour. The median overall survival was >78 months. Fourteen patients had a first primary tumour other than STS. The second tumours (mainly STS) appeared within 0 to 27 years . The median overall survival of the 14 patients in this group from the diagnosis of the first tumour was >102 months. An updated series will be presented.
*Conclusion:* The phenomenon of two or three primary neoplasms in patients in whom one of the tumours was STS occurs in a rate of 7.5% – a significantly higher rate than the occurrence of STS among the general cancer population (1%). Most of the cases occur incidentally. The clinical implications are the need to search for an occult second primary in patients with STS, as an integral part of their follow-up. It is especially true in patients with primary MFH who show a risk for developing a renal-cell-carcinoma.

